# Revisiting the Link between Job Satisfaction and Life Satisfaction: The Role of Basic Psychological Needs

**DOI:** 10.3389/fpsyg.2017.00680

**Published:** 2017-05-09

**Authors:** Wenceslao Unanue, Marcos E. Gómez, Diego Cortez, Juan C. Oyanedel, Andrés Mendiburo-Seguel

**Affiliations:** ^1^Business School, Universidad Adolfo IbáñezSantiago, Chile; ^2^School of Psychology, Universidad Adolfo IbáñezSantiago, Chile; ^3^Facultad de Educación, Universidad Andres BelloSantiago, Chile

**Keywords:** job satisfaction, life satisfaction, need satisfaction, self-determination theory, longitudinal analyses, Chile

## Abstract

The link between job satisfaction and life satisfaction has been extensively explored in the relevant literature. However, the great majority of past research has been carried out using cross-sectional analyses, and almost exclusively in the Western world. Moreover, the underlying psychological mechanisms explaining the link are not yet completely understood. Thus, we report the first research to date which uses both cross-sectional and longitudinal data among workers in Chile—a fast-developing Latin American economy—and which aims to tackle previous limitations. Three studies consistently support a positive link between the constructs. Study 1 (*N* = 636) found that higher job satisfaction predicted higher life satisfaction both contemporaneously and longitudinally, and vice versa, above and beyond several key control variables. Study 2 (*N* = 725) and Study 3 (*N* = 703) replicated Study 1 results, but tested for the first time the role of satisfaction of basic psychological needs (as stated by self-determination theory) in the job–life satisfaction link. This is the most novel contribution of our paper. Key implications not only for individual quality of life, but also for companies' human resource practices emerge from our findings.

## Introduction

How related are job satisfaction and life satisfaction? This question has been extensively explored in the literature (Heller et al., 2002). The spillover hypothesis (Bowling et al., 2010) is the most supported hypothesis to date. It argues that “job experiences spill over onto other spheres of life, and vice versa, suggesting that a positive relationship exists between the two variables” (Heller et al., 2002, p. 816). However, there are still three important research gaps in this area. First, previous research has reported results mainly from the Western world (Rain et al., 1991; Heller et al., 2002; Diener and Tay, 2012). Second, most research in the subject is correlational, which does not allow for the inferring of causality (Bowling et al., 2010). Third, and finally, the lack of research aiming to give a solid theoretical explanation for the results is surprising (Rain et al., 1991; Judge and Watanabe, 1993; Rode, 2004). In fact, the underlying psychological mechanisms explaining the hypothesis are still not completely understood.

Further, aiming to tackle previous limitations, we explored three large samples of workers in Chile (a fast-developing Latin American economy) using both cross-sectional and longitudinal data, which is very rarely done in this field (Rode, 2004). In addition, because recent research has argued that the link between job satisfaction and life satisfaction might be spurious, and that a third variable could be involved (Judge and Watanabe, 1993; Rode, 2004), we tested for the first time the mediational role of basic need satisfaction (Deci and Ryan, 2000) in the job-life satisfaction link.

### Job satisfaction and life satisfaction

Happiness has been conceptualized from the hedonic and eudaimonic approaches (Delle Fave et al., 2011). The hedonic approach defines happiness in relation to the attainment of pleasure and the avoidance of pain (Ryan and Deci, 2001). From this point of view, happiness is often called subjective well-being (Diener, 1984), which consists of cognitive (life satisfaction) and emotional (positive and negative emotions) experiences. Life satisfaction represents the judgment that a person makes about his/her life in several domains (Diener, 1984; Diener and Tay, 2012), and it is the most extended construct for assessing subjective well-being (Helliwell et al., 2013). A growing body of research has shown that higher life satisfaction is associated with several desirable companies' results, such as higher career satisfaction, organizational commitment, and especially, job satisfaction (Diener and Tay, 2012).

Job satisfaction is a key construct in industrial and organizational psychology, and has been associated with multiple desirable outcomes such as job performance, organizational citizenship behavior, absenteeism, and life satisfaction (Heller et al., 2002; Erdogan et al., 2012). Most definitions of job satisfaction tend to focus on how employees feel and think about their work (Locke, 1969; Smith et al., 1969; Weiss, 2002; Drafke, 2009). These definitions, in a very similar way to those of life satisfaction, involve emotional states, feelings, affective responses, and cognitive evaluations of work (Alonso, 2006).

Three hypotheses have been argued to explain the link between job satisfaction and life satisfaction: the segmentation, compensation, and spillover hypotheses (Rain et al., 1991; Heller et al., 2002; Bowling et al., 2010).

### The segmentation hypothesis

The segmentation hypothesis suggests that there is no relationship between job satisfaction and life satisfaction. Theoretical positions such as *partial inclusion* have been proposed to explain the link between both concepts from this perspective (for a review see Rain et al., 1991). However, only one study supported this hypothesis in the meta-analysis by Rain et al. (1991). Indeed, Gupta and Beehr (1981) found more support for the segmentation hypothesis than for the compensation and spillover ones, among 651 employees of five Midwestern organizations. According to the authors, these findings show that work (e.g., job satisfaction) and non-work aspects (e.g., life satisfaction) load on separate factors.

### The compensation hypothesis

The compensation hypothesis states that people compensate for their job dissatisfaction by finding more satisfaction in other areas of their life, and vice versa (Iris and Barrett, 1972). Thus, a negative relationship is postulated. Theoretical positions such as the *principle of substitution* and *catharsis theory* have been proposed to explain this hypothesis (Rain et al., 1991). Very few studies, however, have supported it (e.g., Champoux, 1980; Chacko, 1983; Schlenker and Gutek, 1987; for a review see Rain et al., 1991). For example, Schlenker and Gutek (1987) studied 132 government-employed social workers. Half of them were reassigned to non-professional jobs, and this allowed understanding the impact of their dissatisfaction with the new job on their life satisfaction. The authors found that their discontent because of their work role-loss focused on their new jobs instead of on their satisfaction with life, thus supporting the compensation hypothesis. Chacko (1983) studied a US national probability sample, which was representative of the American labor force. The authors found that satisfaction with working conditions was a negative source of life satisfaction, thus also supporting the compensation hypothesis.

### The spillover hypothesis

The spillover hypothesis argues that there is a positive relationship between job satisfaction and life satisfaction. Theoretical explanations such as *generalization* of belief and attitudes, *conditioning*, and *cognitive dissonance* have been given to explain it (Tait et al., 1989; Rain et al., 1991). To date, the spillover hypothesis is by far the most supported one in the literature. Meta-analytic techniques and literature reviews confirm these findings (Rice et al., 1980; Tait et al., 1989; Rain et al., 1991; Bowling et al., 2010). For example, a meta-analysis by Rice et al. (1980) explored 350 associations between job satisfaction and overall life satisfaction reported in 23 studies. More than 90% of the 350 statistical relationships supported a positive correlation. However, the reported zero-order correlations were mostly small (mid −0.30 for males and mid –0.20 for females), and none of the negative correlations they found was statistically significant.

The Tait et al. (1989) meta-analysis also confirmed that the spillover hypothesis is the most evidence-backed one. They explored 34 studies assessing the link between job and life satisfaction, but the authors found larger correlations than Rice et al. (1980). The corrected correlation (for both sampling error and measurement error) was 0.44. Importantly, whereas the corrected correlation was greater for men (*r* = 0.40) than for women (*r* = 0.20) in studies prior to 1974, the difference between men (*r* = 0.37) and women (*r* = 0.39) disappeared in studies after that year. Demographic changes among women, and the role of work in their lives, may explain these findings.

### The causal direction of the link between job satisfaction and life satisfaction

Despite the strong empirical support for the spillover hypothesis, most research to date is correlational in nature, which does not allow for the inferring of causality. Does life satisfaction predict job satisfaction or vice-versa? Would a bi-directional link be possible? Unfortunately, only a few studies to date have explored cause–effect patterns, and even these have mostly done so under the assumption that either job satisfaction or life satisfaction are dependent variables. For example, whereas Schmitt and Mellon (1980) showed that only life satisfaction predicts job satisfaction, Orpen (1978) and Chacko (1983) showed that only job satisfaction causes life satisfaction (Judge and Watanabe, 1993). Thus, there are hardly any studies that explore and show a reciprocal link (Rain et al., 1991; Judge and Watanabe, 1993). Nonetheless, a bi-directional link may be expected, thus making previous results inconsistent with the theory.

#### The causal influence of life satisfaction in job satisfaction: the top-down model

The link between job satisfaction and life satisfaction can be interpreted in two ways: top-down vs. bottom-up models. The top-down model offers a dispositional explanation, claiming that “basic differences in personality and affectivity predispose people to be differentially satisfied with various aspects of their lives, including their jobs” (Heller et al., 2002, p. 816). Thus, affective states spillover into people's evaluations of their jobs (Judge and Watanabe, 1993, p. 939). In fact, a substantial body of research has shown that higher life satisfaction is associated with several desirable results for companies, such as higher career satisfaction, organizational commitment, and—of interest here—job satisfaction (Diener and Tay, 2012).

#### The causal influence of job satisfaction on life satisfaction: the bottom-up model

Job satisfaction is a key indicator of workers' well-being (Diener and Tay, 2012), and therefore can also influence life satisfaction (Judge et al., 2001). Thus, the bottom-up model suggests a situational explanation. That is, “because the job is an important part of adult daily life, people who enjoy their jobs will report greater overall satisfaction with their lives” (Heller et al., 2002, p. 816). Indeed, research has consistently found that higher job satisfaction is associated with higher life satisfaction (Rice et al., 1980; Rain et al., 1991). This causal influence of job satisfaction on life satisfaction reflects the importance of work in people's lives, and is the most hypothesized direction of the link (Judge and Watanabe, 1993).

#### The bi-directional link between job satisfaction and life satisfaction

The relationship between job and life satisfaction is likely not only to be a one-way direction, but also reciprocal. Thus, a bi-directional link may be expected (Heller et al., 2002). However, only a few exceptions have explored and found reverse causality (Keon and McDonald, 1982; Judge and Watanabe, 1993). For example, Keon and McDonald (1982) found that job satisfaction and life satisfaction were jointly determined among employees of an auto parts manufacturer in the US. Judge and Watanabe (1993) tested a causal model exploring bi-directional associations between job and life satisfaction, over a 5-year period, and controlling for several exogenous influences on life satisfaction (such as age, gender, education, wage rate, and marital status). Based on a national probability sample of US workers, they found that job and life satisfaction were significantly and reciprocally related both cross-sectionally and longitudinally. However, due to the small amount of research on this, and various limitations in the previous studies, scholars have advocated for more longitudinal research in the field exploring causality and the bi-directional link (Judge and Watanabe, 1993; Rode, 2004). Finally, the meta-analysis by Bowling et al. (2010) explored longitudinally the association between job satisfaction and subjective well-being. Despite the authors having found a reciprocal causal link between the constructs, the subjective well-being measure did not assess life satisfaction independently. The composite subjective well-being measure included not only life satisfaction, but also positive affect, happiness, and negative affect.

### Individual differences in the link between job satisfaction and life satisfaction: moderators and mediators

To date, the great majority of previous research has supported the spillover hypothesis. However, what are the underlying psychological processes behind the link between job satisfaction and life satisfaction? Unfortunately, most studies have not advocated a theoretical proposition (Kabanoff, 1980; Rain et al., 1991), and the three hypotheses approach seems too simplistic (Rain et al., 1991). Indeed, researchers have suggested that more than one relationship between job satisfaction and life satisfaction may operate at any given time point, and that the three hypotheses “may exist for different individuals” (Heller et al., 2002, p. 816). Thus, more studies exploring different mediators and moderators are needed (Rice et al., 1980; Tait et al., 1989; Rain et al., 1991). Initially, the focus was on the moderator role of gender (Tait et al., 1989; Rain et al., 1991). However, other potential moderators (such as age, self-employment, locus of control, importance of work, need for achievement) have been suggested later on, but either they are not all consistent or they have not been tested yet (for a review see Rain et al., 1991).

Nobel research has proposed another look at the job–life satisfaction link. For example, it has been suggested that “much of the relationship between job satisfaction and life satisfaction is spurious, resulting from common influences” (Rode, 2004, p. 1206). Therefore, a third or confounding or mediator variable could be involved (Heller et al., 2002). Following this theorization, Heller et al. (2002) explored the role of personality (Big Five, positive/negative affectivity, and core self-evaluations) in the link between job satisfaction and life satisfaction. Using a longitudinal design among US employees, and multisource data, the authors found support for the confounding role of personality, especially core self-evaluations (neuroticism, locus of control, self-esteem, and generalized self-efficacy). After controlling for personality, the magnitude of the association between job and life satisfaction decreased significantly, suggesting the presence of a third variable involved.

However, despite the importance and novelty of these results, they need to be treated with caution, due to some limitations. First, despite the fact that Heller et al. (2002) argued that their design was longitudinal, it is correlational in nature. A suitable longitudinal model needs to include all relevant T1 and T2 variables in the same model and modeling the stability paths, which in their study was not the case. Second, the authors did not test reciprocal causality: in their first two models, they assumed that job satisfaction precedes life satisfaction, while in the latter two models they took the opposite approach, meaning that life satisfaction precedes job satisfaction.

In another study, Rode (2004) replicated the findings of Heller et al. (2002). Using a nationally representative US sample, the author tested a comprehensive model examining the relationship between job satisfaction, life satisfaction and core self-evaluations and found a positive relationship between job satisfaction and life satisfaction over time. Interestingly, the link became non-significant after controlling for the effects of core self-evaluations. Similarly to Heller et al. (2002), Rode's (2004) findings present some limitations. First, the author only measured life satisfaction at time 1 (T1) and core self-evaluations at time 2 (T2). Therefore, although he argued the model is a longitudinal cross-lagged one, it is only cross-sectional in nature. A suitable cross-lagged model needs to include lagged paths from each relevant measure (e.g., life satisfaction, job satisfaction) at T1 to all relevant measures at T2, controlling for stability effects (i.e., all constructs need to be represented as potential antecedents and as potential consequences of all other constructs).

Previous results have given initial empirical support to the idea that the link between job satisfaction and life satisfaction may be spurious, and that a third variable could be involved. However, those studies only focused on personality and affectivity variables, without exploring other constructs. We hypothesize that the satisfaction of basic psychological needs as proposed by self-determination theory (Deci and Ryan, 2000) may also play a key role in the job–life satisfaction link. Initial evidence may be found in Hombrados-Mendieta and Cosano-Rivas (2013) and Di Fabio and Kenny (2016a). Hombrados-Mendieta and Cosano-Rivas (2013) found that workplace support (a proxy of the need for relatedness) protects job satisfaction and life satisfaction against the negative effects of burnout. Di Fabio and Kenny (2016a) stated that the need for relationship and the need for self-determination (a proxy of the need for autonomy) are crucial for workers' well-being. These preliminary results allow us to hypothesize that need satisfaction may mediate the relationship between job satisfaction and life satisfaction.

### The role of basic psychological needs in the job–life satisfaction link

Self-determination theory (Deci and Ryan, 2000) is a “macro theory of human motivation, emotion and personality” (Vansteenkiste et al., 2010, p. 105). It states that human beings have three psychological needs—autonomy, competence, and relatedness—which are essential for psychological well-being and integration. Just as plants need essential nutrients, such as water and minerals, so people have essential needs too (Reis et al., 2000).

The need for *autonomy* (DeCharms, 1968) refers to the perception that our behavior is volitional and meaningful; the need for *competence* (White, 1959) refers to feeling effective and efficient in our behavior, as well as being able to successfully manage difficult challenges and meet performance standards; the need for *relatedness* (Baumeister and Leary, 1995) refers to feeling connected, appreciated and understood by others who are important (Vansteenkiste et al., 2010; Unanue et al., 2014). Therefore, feeling able to decide what to do and that these actions are valuable and enjoyable (*autonomy*); feeling good at daily activities (*competence*); and having meaningful and deep relationships with people who is important to us (*relatedness*) are the key nutrients for people flourishing (Deci and Ryan, 2000).

A substantial amount of research has supported the self-determination theory claims, showing that satisfaction of psychological needs is significantly associated with higher well-being both in general life and at work. For example, in everyday life settings, Unanue et al. (2014) showed that higher need satisfaction *in life settings* is associated with higher well-being (e.g., life satisfaction, vitality and positive affect) and lower ill-being (physical symptoms, negative affect and depressive symptoms). These findings have been replicated across the lifespan, as well as cross-culturally (Chen et al., 2014). In job settings, a recent meta-analysis (Van den Broeck et al., 2016), reviewing 99 studies with 119 distinct samples, found that the satisfaction of basic psychological needs *at work* is significantly associated not only with higher well-being (e.g., life satisfaction) but also with several desirable organizational outcomes (e.g., job satisfaction).

Previous findings allow us to hypothesize that the link between job satisfaction and life satisfaction may be spurious, and instead is rooted in basic needs satisfaction. This is because both job satisfaction and life satisfaction seem to be dispositionally based, and the same characteristics that predict one construct (job satisfaction) also predict the other (life satisfaction) (Heller et al., 2002). The role of psychological needs was suggested more than 30 years ago (Champoux, 1981; Rain et al., 1991). However, it has not been tested until now.

Therefore, in our research, we explore the confounding influence of basic need satisfaction (Deci and Ryan, 2000) on the link between job and life satisfaction. Because need satisfaction in life settings as well as at work predict well-being, our research will test both kinds of need satisfaction in the mentioned link.

### Research gaps: justification for the present research

As mentioned before, meta-analytic techniques and literature reviews confirm that the spillover hypothesis is the most supported explanation for the link between job and life satisfaction. However, several methodological and theoretical research gaps have emerged in the field (Tait et al., 1989; Rain et al., 1991; Heller et al., 2002).

In methodological terms, there are two aspects that need to be improved: samples and design (Rain et al., 1991). First, most research so far has focused on samples from the Western world^1^. Therefore, culture has not been taken into account. Nonetheless, cross-cultural research has shown that cultural aspects might influence several links (Thomas and Au, 2002; Thomas and Pekerti, 2003). Moreover, recent research has shown that the effects of happiness (such as life satisfaction) may be specific to some individualistic cultures (Ford et al., 2015). Such findings raise the question of whether the associations between job and life satisfaction found in the Western world are also held in different populations with different demographic characteristics, like Chile (Heller et al., 2002).

Second, most designs are still correlational in nature, and reciprocal causality has not been extensively explored using suitable cross-lagged models (Rain et al., 1991; Judge and Watanabe, 1993; Rode, 2004). In theoretical terms, and in spite of the spillover hypothesis receiving most of the empirical support, no satisfactory theoretical explanation has been offered (Rain et al., 1991). Indeed, research so far has done little to test the underlying psychological process behind the link (Rain et al., 1991; Judge and Watanabe, 1993).

#### Job satisfaction research in Chile

Data on well-being has only appeared recently in Latin America (Montero and Vásquez, 2015), and there is only one paper in which the job-life satisfaction link in Chile is studied: Loewe et al. (2014). Loewe et al. (2014) found support for the spillover hypothesis, showing that Chilean workers attribute most importance to their financial “situation, followed by family, work, and health” (p. 80). However, several limitations emerge from the paper. For example, it is correlational in nature and neither alternative models nor causality was tested. In addition, no underlying mechanisms for explaining the job-life satisfaction link were tested.

A few additional researchers have explored job satisfaction in Chile. For example, Cassar (2010) studied a representative sample of the Oxford Poverty and Human Development Initiative (OPHI) database, and found that the degree of employment protection, workplace facilities and level of independence were positively associated with job satisfaction. Using the same data set, Montero and Rau (2014) found a positive relationship between salaries and job satisfaction, while Montero and Vásquez (2015) showed the impact of reference wages on job satisfaction. Finally, using the CASEN survey (National Socio-Economic Characterization Survey) and OPHI, Montero and Rau (2015) found that part-time work has a negative effect on job and life satisfaction for men, but a positive effect for women. The above-mentioned represent a notable contribution to the job satisfaction literature in Chile. Nonetheless, these studies suffer from certain limitations. They are cross-sectional in nature, they did not explore the underlying psychological process, and more importantly, they did not test the link between job satisfaction and life satisfaction.

### Summary of aims and predictions

With the aim of tackling previous research gaps, we conducted three different studies among Chilean workers. We had three objectives in mind. First, to test whether the cross-sectional positive link (spillover hypothesis) between job and life satisfaction found in the Western world is held among workers from Chile, controlling for several key confounding variables not assessed to date. Second, because the great majority of past research has been carried out using cross-sectional analyses, we used longitudinal designs to test a possible bi-directional causal link between the core variables. Third, and finally, for the first time a comprehensive model was tested. It allowed us to examine the relationship between job and life satisfaction over time while taking into account the possible confounding role of a third variable: basic psychological needs.

Chile is a country with increasing mental health problems at work (MINSAL, 2017). Indeed, the Ministry of Labour in Chile warns that Chilean employees are in danger of serious psychological problems at work (Mutual de Seguridad, 2015). By understanding the job-life satisfaction dynamic we aim to help companies to develop strategies not only for protecting their employees' mental health, but also for improving employees' quality of life and happiness. In addition, because of the close link between life/job satisfaction and productivity, our findings may help to improve companies' profitability and sustainability (Diener and Tay, 2012; Montero and Vásquez, 2015).

Further, based on the previous evidence, we tested the following hypotheses:

(H1) Job satisfaction is positively associated with life satisfaction correlationally (Study 1; Study 2; Study 3).
(H2) Higher job satisfaction predicts higher life satisfaction prospectively and vice versa (longitudinally) (Study 1; Study 2; Study 3).
(H3) Need satisfaction *at work* explains the positive link between job satisfaction and life satisfaction both contemporaneously (correlationally) and prospectively (longitudinally). (Study 2).
(H4) Need satisfaction *in life* explains the positive link between job satisfaction and life satisfaction both contemporaneously (correlationally) and prospectively (longitudinally). (Study 3).

## Study 1

### Method

#### Procedure

Study 1 was carried out in accordance with the guideline recommendations of the American Psychological Association, British Psychological Society and World Medical Association Declaration of Helsinki. All subjects gave written informed consent in accordance with the Declaration of Helsinki. The protocol was approved by the Ethics Committee of the Adolfo Ibáñez University.

At baseline (June 2016; T1), participants were invited online to take part in a research project where the core measures for the present paper were collected (job satisfaction and life satisfaction)^2^. Two months later (August 2016; T2), the same participants were asked to complete another online survey with identical measures. Initially, respondents were sent an introductory email containing a brief description of the study, along with a web link to the survey created using Qualtrics software.

Following Simmons et al.'s (2011) guidelines, all target sample sizes were determined in advance in Study 1, and in all further studies. Using a power analysis with G^*^Power 3.1 (Faul et al., 2009), considering a hypothesized small effect size (0.18; Bosco et al., 2015), power of 0.80, and *p*-value = 0.05, the desired sample was estimated as a minimum of *N* = 187. Both our cross-sectional data at T1 (*N* = 636) and T2 (*N* = 268), and our longitudinal data (*N* = 210), fulfill the required sample size. This minimum sample size (*N* = 187) also applies to Study 2 and Study 3.

We followed a key rule for collecting T1 data. We informed participants that the online system would be open for only 3 days (due to the original design of Unanue et al., 2017). Participants were informed that the project was part of a longitudinal study and were asked for their consent in participating in future waves. All T1 participants were sent a further email in August 2016 (T2). The rule for collecting T2 data was only slightly different. To be able to find reasonable effect sizes, we considered that it would be important to recruit a large sample aiming to reach a power of 0.80 both cross-sectionally and longitudinally.

Almost all constructs used in Study 1 showed acceptable distributions (George and Mallery, 2010). Skew values were acceptable for life satisfaction (T1: −1.45; T2: −0.05) and job satisfaction (T1: −1.2; T2: −1.03). Kurtosis values were acceptable for job satisfaction (T1: 1.75; T2: 1.34). In the case of life satisfaction, Kurtosis values were acceptable at T2 (0.58), but not at T1 (4.41).

#### Sample

In total, 636 Chilean working adults (52.8% female) aged from 22 to 71 years (Mean age = 39.76; *SD* = 8.61) finished the whole survey and completed our T1 measures. Of them, 475 (74.69%) provided their e-mail address for future waves. At T2, 268 participants (55.6% female) aged from 20 to 80 years (Mean age = 40.47; *SD* = 10.73) answered our core measures (56.42% response rate). Finally, 210 workers (56.2% female) aged from 23 to 71 years (Mean age = 40.16; *SD* = 9.17) answered both waves.^3^

#### Measures

We used the following measures for Study 1.

##### Job satisfaction

We used a single question: “All in all, how satisfied are you with your job?” Participants answered on an 11-point scale, ranging from “extremely unsatisfied” (0) to “extremely satisfied” (10). Meta-analytic analysis demonstrated that this single question is highly valid for measuring job satisfaction (Dolbier et al., 2005).

##### Life satisfaction

We used a single question: “All in all, how satisfied are you with your life?” Participants answered on an 11-point scale, ranging from “extremely unsatisfied” (0) to “extremely satisfied” (10). This question is the most extensive single item question designed to measure life satisfaction, and it has shown good psychometric properties (Helliwell et al., 2013).

##### Demographic variables

At T1, we asked participants to report their age and gender status. However, at T2, we asked participants also to report additional demographic variables such as education, economic sector (mining, transport, etc.), working area (marketing, finance, etc.), managerial functions (whether or not they lead people), monthly personal income, and monthly family income. Our results showed that participants who answered both waves differed in several demographic characteristics such as education (high school education 5.71%, Bachelors degree 31.90%, post-graduate degree 61.43%, other 0.95%); economic sector (health and social services 18.10%, education 14.76%, commerce 7.62%, community services 5.71%, public sector 4.76%, mining 4.29%, financial services 3.81%, manufacturing 3.81%, transport 3.81%, others 33.33%); working area (accounting 0.95%, technology 1.90%, administration 4.29%, finance 5.71%, operations 7.14%, marketing 6.19%, human resources 36.20%, others 37.62%); managerial positions (58%); net personal monthly average income (mean = US$ 5,789.30; *SD* = 3,664.30); and net family monthly average income (US$1,853.10; *SD* = 1,396.30). As stated by Judge and Watanabe (1993), what is crucial when studying the link between job and life satisfaction is “the use of extensive controls derived from past theory and research”, the lack of which was a limitation in previous studies (p. 940). Thus, we aimed to tackle this limitation by using our extensive number of control variables.

### Results

#### Data analysis

All constructs of interest were measured at T1 and T2. Descriptive statistics and intercorrelations for all the study variables are shown in Table 1 (cross-sectional analyses) and Table 2 (longitudinal analysis). We used AMOS 22 software (Amos Development Corporation, Florida, USA) to estimate an autoregressive cross-lagged model (Finkel, 1995) through path analysis. We used full maximum likelihood estimation in all our analyses. All the paths in Study 1 and all further studies were standardized. Because this was a saturated model, the fit indices were perfect.

**Table 1:** **Descriptives and inter-correlations between all study 1 variables**.

	**Scale range**	***M***	***SD***	**1**	**2**	**3**	**4**
**TIME 1**							
1. Gender		1.53	0.50				
2. Age		39.76	8.61	−0.12^**^			
3. Life Satisfaction	0–10	8.14	1.47	0.04	0.12^**^		
4. Job Satisfaction	0–10	7.60	2.01	0.04	0.22^**^	0.45^**^	
**TIME 2**							
1. Gender		1.56	0.50				
2. Age		40.47	10.73	−0.22^**^			
3. Life Satisfaction	0–10	8.10	1.19	−0.02	0.14^*^		
4. Job Satisfaction	0–10	7.44	1.82	−0.03	0.21^**^	0.50^**^	

* p < 0.05;

** *p < 0.01*.

**Table 2:** **Descriptives and inter-correlations between all study 1 variables, at Time 1 (T1) and Time 2 (T2) (longitudinal data)**.

	**Scale range**	***M***	***SD***	**1**	**2**	**3**	**4**	**5**	**6**	**7**	**8**	**9**	**10**	**11**	**12**
1. Gender		1.56	0.50												
2. Age		40.16	9.17	−0.13											
3. Education		5.30	0.96	−0.13	−0.02										
4. Managerial functions		1.42	0.50	0.16^*^	−0.26^**^	−0.04									
5. Working area		5.72	2.17	0.07	0.15^*^	−0.01	0.17^*^								
6. Economic sector		15.98	5.11	0.22^**^	0.01	0.09	0.06	0.10							
7. Monthly personal income (US$)		3401.66	2878.96	−0.18^*^	0.42^**^	0.04	−0.35^**^	−0.10	−0.12						
8. Monthly family income (US$)		5789.29	3664.34	0.08	0.18^*^	0.05	−0.32^**^	−0.05	−0.04	0.62^**^					
9. Life Satisfaction T1	0–10	8.33	1.14	0.04	0.21^**^	−0.09	−0.10	0.03	0.03	0.10	0.18^*^				
10. Job Satisfaction T1	0–10	7.66	1.89	0.01	0.18^**^	−0.04	−0.12	0.00	0.05	0.19^**^	0.25^**^	0.49^**^			
11. Life Satisfaction T2	0–10	8.19	1.12	0.09	0.07	−0.06	−0.17^*^	0.02	0.00	0.15^*^	0.19^*^	0.56^**^	0.43^**^		
12. Job Satisfaction T2	0–10	7.50	1.84	0.02	0.21^**^	−0.03	−0.21^**^	0.04	0.02	0.15^*^	0.19^*^	0.46^**^	0.58^**^	0.52^**^	

* p < 0.05;

** *p < 0.01*.

##### Cross-sectional analyses

**Figure 1:**
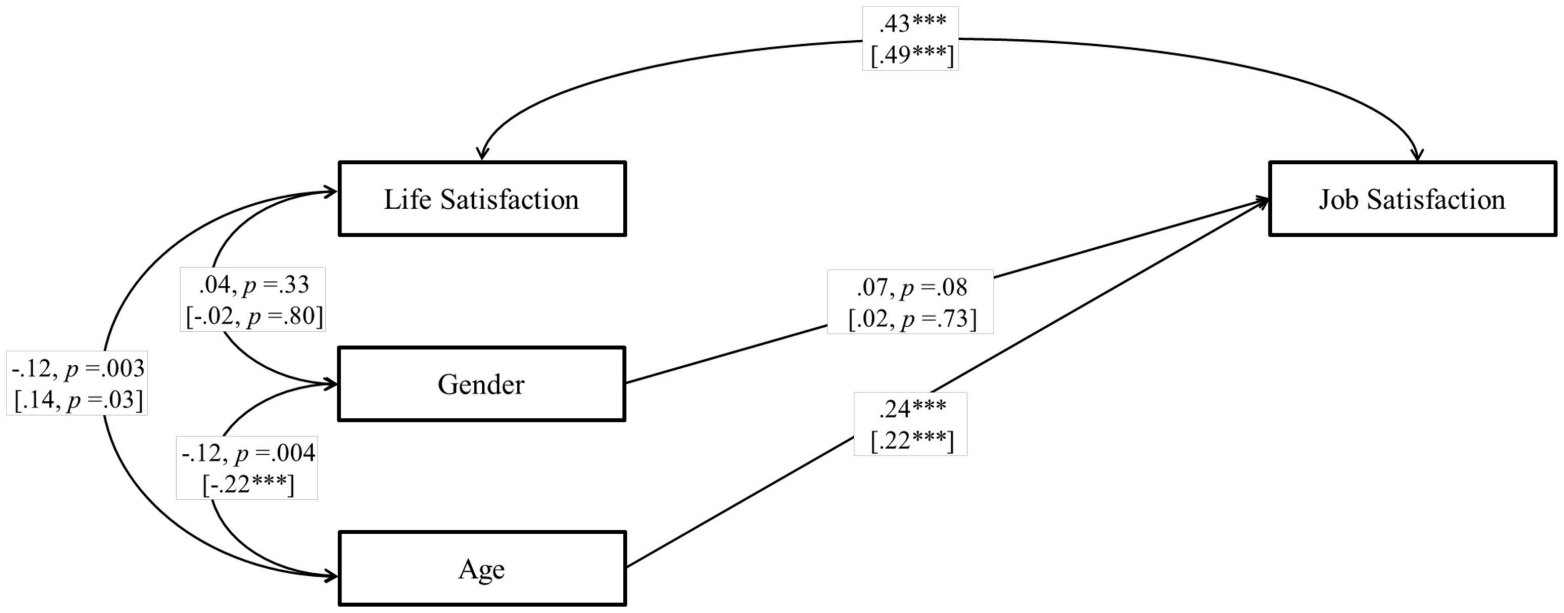
**Study 1**. Structural correlational model for the association between job satisfaction and life satisfaction at Tl and T2. T2 data are in brackets. Coefficients shown are standardized paths.T1, Time 1; T2, Time 2. ^***^p < 0.001.

First, we set up a cross-sectional model to test H1, and to determine the size of the association between job satisfaction and life satisfaction at T1 and T2. We controlled for age and gender. At T1 (Figure 1), we found that job satisfaction was significantly and positively related to life satisfaction, β = 0.43, *p* < 0.001. At T2 (Figure 1), job satisfaction was also significantly and positively related to life satisfaction, β = 0.49, *p* < 0.001. These results show a medium effect size (Cohen, 1992) for the association between job and life satisfaction, supporting H1 at both T1 and T2.

##### Longitudinal analysis

**Figure 2:**
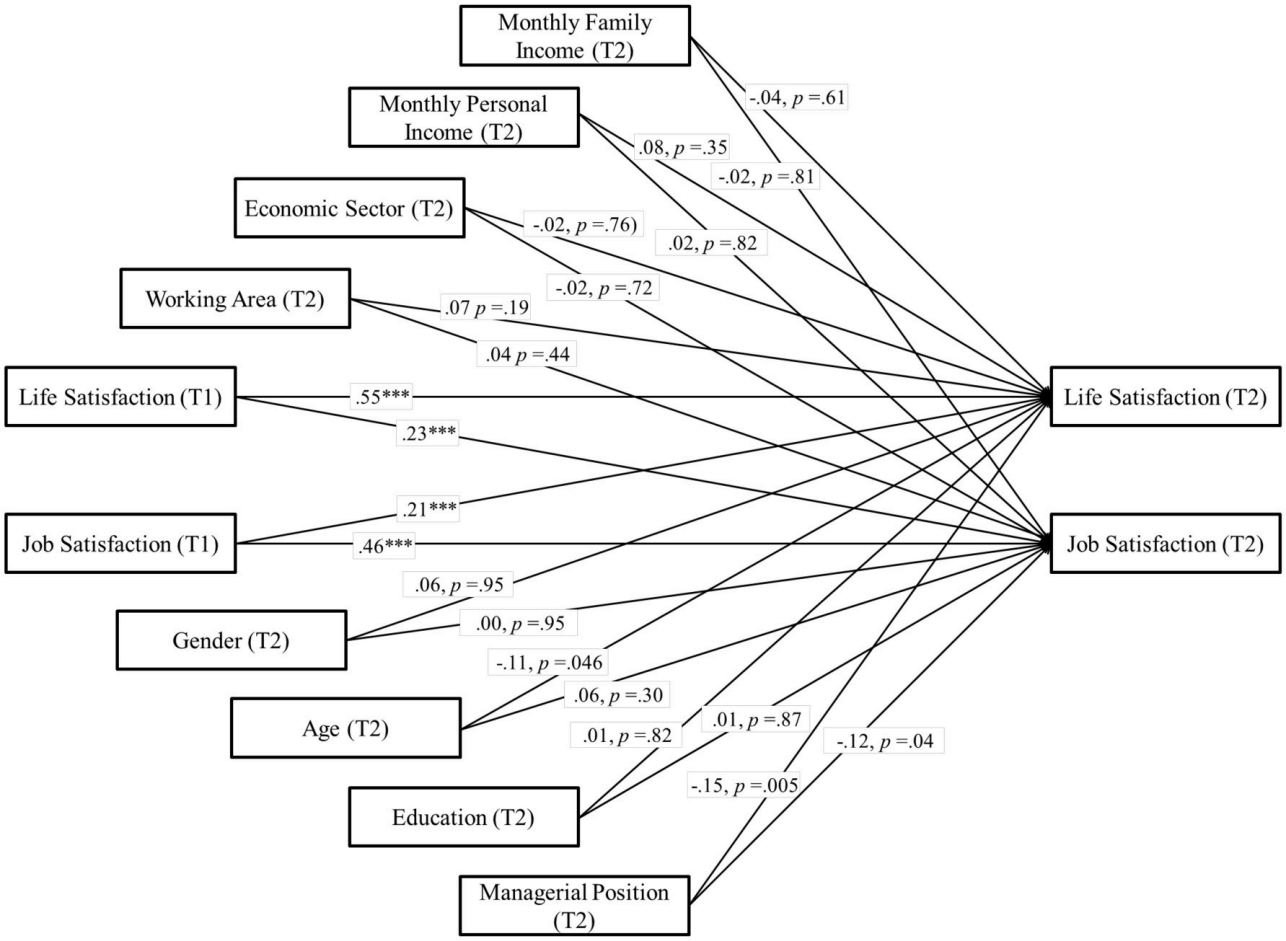
**Study 1**. Structural longitudinal model for the association between job satisfaction and life satisfaction. Coefficients shown are standardized paths. Error terms and covariances are not shown to enhance visual clarity. Tl, Time 1; T2, Time 2. ^***^p < 0.001.

We used a cross-lagged longitudinal design to disentangle the causal direction between job satisfaction and life satisfaction, aiming to test H2 and H3. Here, we controlled for age and gender, but also for education, economic sector, working area, managerial functions, net monthly personal income, and net monthly family income. A two-factor model where each T2 measure was regressed on both its own lagged measure as well as the other lagged measures was defined. We allowed the observed measures to covary within each time point. Thus, all constructs were represented as potential antecedents and as potential consequences of all other constructs, while controlling for stability effects. Values of *R*^2^ were also acceptable (*p* < 0.001) ranging from 0.49 (life satisfaction) to 0.43 (job satisfaction) (*p* < 0.001). We found that job satisfaction was a significant and positive prospective predictor of life satisfaction, β = 0.21, *p* < 0.01. In addition, we found that life satisfaction was a significant and positive prospective predictor of job satisfaction, β = 0.23, *p* < 0.001. These results show that longitudinally, job satisfaction predicts life satisfaction and vice versa, supporting H2. Importantly, the prospective bi-directional link remained above and beyond all our control variables. Paths are reported in Figure 2.

### Study 1 brief discussion

Study 1 supported H1 and H2, but also allowed us to tackle several limitations found in the previous literature. Supporting the spillover hypothesis (Tait et al., 1989; Rain et al., 1991; Bowling et al., 2010), we found a cross-sectional and causal positive bi-directional link between job satisfaction and life satisfaction among workers from Chile. Importantly, the correlational and longitudinal links were found while controlling for several demographic and confounding variables not assessed in previous research (age, gender, education, marital status, salary, etc.). Indeed, Judge and Watanabe (1993) argued that when studying the link between job and life satisfaction, the use of extensive controls is crucial. Indeed, our results suggest taking into account the demographic variables as control variables when studying the job-life satisfaction link. Finally, it is worth mentioning that a limitation of Study 1 design is that it did not allow testing a third variable that may be driving the mentioned link. Thus, in Study 2 we tested the confounding influence of need satisfaction at work, whereas in Study 3 we tested the confounding influence of need satisfaction in general life setting.

## Study 2

### Method

#### Procedure

Study 2 followed the same ethical rules as Study 1. We utilized a cross-lagged longitudinal design with 4 weeks between the two observations and a wide sample of Chilean worker adults. At the baseline (September 2016; T1), participants were invited online to take part in a research project where the core measures for the present paper were collected (job satisfaction, life satisfaction and need satisfaction at work^4^). A university in Santiago provided a mailing list for both Study 2 and Study 3. Participants were informed that the project was part of a longitudinal study and were asked for their consent to participate in future waves. Four weeks later (October 2016; T2), the same participants were asked to complete another online survey with identical measures. Respondents received an introductory email containing a brief description of the study, along with a web link to the survey created using Qualtrics software.

We followed a key rule for collecting T1 and T2 data. The survey was kept open for only 1 week, and every working day a polite reminder was sent to those respondents who had not answered. Both our cross-sectional data at T1 (*N* = 725) and T2 (*N* = 275), and our longitudinal data (*N* = 274) fulfilled the required minimum sample size according to our power analysis mentioned previously.

#### Sample

In total, 725 Chilean worker adults (47.9% female) aged from 21 to 72 years (Mean age = 38.03; *SD* = 10.01) finished the whole survey and completed our T1 measures. At T2, 275 participants (47.3% female) aged from 21 to 72 years (Mean age = 39.69; *SD* = 10.24) answered our core measures (37.93% response rate). Finally, 274 workers (47.1% female) aged from 21 to 72 years (Mean age = 39.75; *SD* = 10.20) answered both waves.

All constructs used in Study 2 showed acceptable distributions (George and Mallery, 2010). Skew values were acceptable for life satisfaction (T1: −1.14; T2: −0.98), job satisfaction (T1: −0.69; T2: −0.91) and need satisfaction at work (T1: −0.53; T2: 0.62). Kurtosis values were acceptable for life satisfaction (T1: 1.53; T2: 0.91), job satisfaction (T1: −0.13; T2: 0.62) and need satisfaction at work (T1: 0.09; T2: −0.04).

#### Measures

Job satisfaction and life satisfaction were measured using the same single items used in Study 1. However, in this study, the job satisfaction question was answered on a scale from 1 to 7. Need satisfaction was measured using the Satisfaction items of the Need Satisfaction and Frustration scales adapted to the work context (Chen et al., 2014). The satisfaction subscale included 12 items: four for autonomy (“I feel my choices on my job express who I really am”), four for competence (“At work, I feel capable at what I do”), and four for relatedness (“At work, I feel connected with people who care for me, and for whom I care”). Participants rated these statements on a 7-point Likert-type scale ranging from *1 (not at all true)* to *7 (very true)*. The internal reliability of the need satisfaction scale was good, both at T1 (α = 0.90) and T2 (α = 0.90). Chen et al.'s (2014) scale has shown good psychometric properties and demonstrated that the items load in a single factor. Thus, we calculated a need satisfaction mean score by averaging its 12 indicators.

### Results

#### Data analysis

All constructs were measured at T1 and T2. Descriptive statistics and intercorrelations for all the study variables are shown in Table 3 (cross-sectional analyses) and Table 4 (longitudinal analysis). We used AMOS 22 software (Amos Development Corporation, Florida, USA) to estimate an autoregressive cross-lagged model through path analysis (Finkel, 1995). We used full maximum likelihood estimation in all our analyses. Because this was a saturated model, the fit indices were perfect.

**Table 3:** **Descriptives and inter-correlations between all study 2 variables**.

	**Scale range**	***M***	***SD***	**1**	**2**	**3**	**4**	**5**
**TIME 1**								
1. Gender		1.48	0.50					
2. Age		38.30	10.01	−0.20^**^				
3. Life Satisfaction	0–10	7.82	1.72	−0.04	0.03			
4. Job Satisfaction	1–7	5.02	1.34	−0.02	0.15^**^	0.30^**^		
5. Need Satisfaction at Work	1–7	5.45	0.94	−0.03	0.14^**^	0.40^**^	0.64^**^	
**TIME 2**								
1. Gender		1.47	0.50					
2. Age		39.69	10.24	−0.15^*^				
3. Life Satisfaction	0–10	7.86	1.53	0.00	0.09			
4. Job Satisfaction	1–7	5.07	1.29	0.00	0.24^**^	0.40^**^		
5. Need Satisfaction at Work	1–7	5.45	0.91	0.00	0.20^**^	0.42^**^	0.73^**^	

* p < 0.05;

** *p < 0.01*.

**Table 4:** **Descriptives and inter-correlations between all study 2 variables, at Time 1 (T1) and Time 2 (T2) (longitudinal data)**.

	**Scale range**	***M***	***SD***	**1**	**2**	**3**	**4**	**5**	**6**	**7**	**8**
1. Gender		1.47	0.50								
2. Age		39.75	10.20	−0.15^*^							
3. Life Satisfaction (T1)	0–10	7.89	1.58	−0.05	0.04						
4. Job Satisfaction (T1)	1–7	5.05	1.30	−0.07	0.27^**^	0.28^**^					
5. Need Satisfaction at Work (T1)	1–7	5.53	0.89	0.02	0.22^**^	0.41^**^	0.59^**^				
6. Life Satisfaction (T2)	0–10	7.86	1.54	−0.01	0.09	0.69^**^	0.28^**^	0.35^**^			
7. Job Satisfaction (T2)	1–7	5.08	1.29	0.00	0.23^**^	0.31^**^	0.61^**^	0.63^**^	0.40^**^		
8. Need Satisfaction at Work (T2)	1–7	5.45	0.91	0.01	0.19^**^	0.37^**^	0.49^**^	0.79^**^	0.42^**^	0.73^**^	

* p < 0.05;

** *p < 0.01*.

##### Cross-sectional analyses

First, we set up a cross-sectional model. At T1 (Supplementary Figure 1), we found that job satisfaction was significantly and positively related to life satisfaction, β = 0.30, *p* < 0.001. Thus, H1 was supported at T1. Then, to test H3, we included need satisfaction in the model. We allowed need satisfaction to predict both job and life satisfaction. We found that need satisfaction was positively associated with both life satisfaction, β = 0.40, *p* < 0.001, and job satisfaction, β = 0.64, *p* < 0.001. In support of H3, when need satisfaction was included in the model, the path between life and job satisfaction becomes non-significant, β = 0.06, *p* = 0.09 (Supplementary Figure 2). At T2 (Supplementary Figure 1), we found that job satisfaction was significantly and positively associated with life satisfaction, β = 0.40, *p* < 0.001. Thus, H1 was also supported at T2. Then, to test H3, we included need satisfaction in the model. We found that need satisfaction was positively associated with both life satisfaction, β = 0.42, *p* < 0.001, and job satisfaction, β = 0.74, *p* < 0.001 (Supplementary Figure 2). In support of H3, when need satisfaction was included in the model, the path between life satisfaction and job satisfaction, although still significant, decreased by almost half of its original value, β = 0.16, *p* < 0.10.

##### Longitudinal analysis

**Figure 3:**
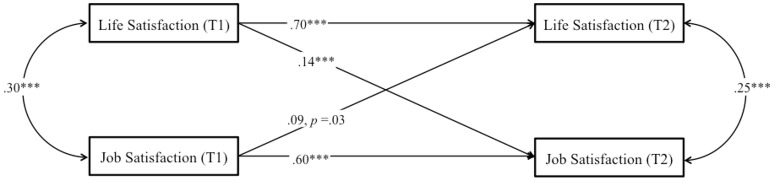
**Study 2**. Structural longitudinal model for the association between job satisfaction and life satisfaction. Coefficients shown are standardized paths. Error terms are not shown to enhance visual clarity. T1, Time 1, T2, Time 2. ^***^p < 0.001.

**Figure 4:**
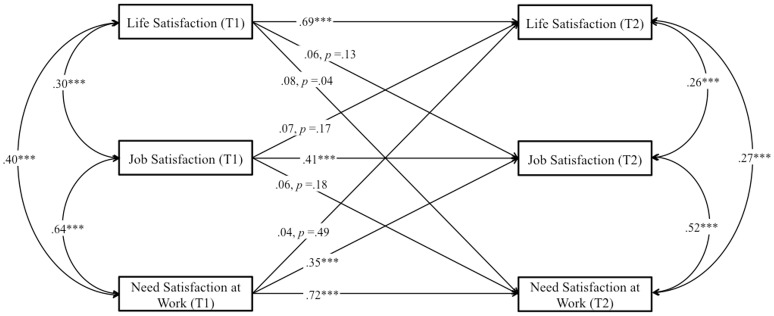
**Study 2**. Structural longitudinal model for the association between job satisfaction and life satisfaction, and need satisfaction at work. Coefficients shown are standardized paths. Error terms are not shown to enhance visual clarity. T1, Ti me 1; T2, Time 2. ^***^p < 0.001.

First, we started with a structural cross-lagged reciprocal model for our core variables (job and life satisfaction), following the same strategy as for Study 1. Second, we allowed the three measures to covary within each time point, and we modeled lagged paths from each of the three measures to all three measures at each successive time point. Therefore, all constructs were represented both as potential antecedents and potential consequences of all other constructs, while controlling for stability effects. Supporting H2, we found that job satisfaction was a positive prospective predictor of life satisfaction, β = 0.09, *p* < 0.05, and that life satisfaction was a significant and positive prospective predictor of job satisfaction, β = 0.14, *p* < 0.001 (Figure 3). *R*^2^ was good for job satisfaction (0.43) and life satisfaction (0.53). Given the important role hypothesized by need satisfaction at work in the job-life satisfaction relationship, we tested a structural model to establish whether need satisfaction influenced both variables (Figure 4). In this new model, *R*^2^ was good for job satisfaction (0.50), life satisfaction (0.53), and need satisfaction at work (0.62). We found that need satisfaction at work was a significant and positive prospective predictor of job satisfaction, β = 0.35, *p* < 0.001 and that life satisfaction was a significant and positive prospective predictor of need satisfaction at work, β = 0.08, *p* < 0.05. They were the only significant paths. Importantly, after controlling for need satisfaction at work, the reciprocal link between job satisfaction and life satisfaction becomes non-significant, supporting H3. Indeed, job satisfaction did not predict life satisfaction longitudinally, β = 0.07, *p* = 0.17 and similarly, life satisfaction did not predict job satisfaction longitudinally, β = 0.06, *p* = 0.13 when controlling for need satisfaction at work.

### Study 2 brief discussion

The results of Study 2 replicated the findings of Study 1, thus supporting H1 and H2, and, in turn, the spillover hypothesis (Tait et al., 1989; Rain et al., 1991). More importantly, taking a bottom-up approach (Diener and Tay, 2012), we found that the cross-sectional and longitudinal links between job satisfaction and life satisfaction was mediated by need satisfaction at work (Deci and Ryan, 2000). This key finding supports H3. However, despite the important results of Study 2, it only tested the confounding role of need satisfaction in job settings. Thus, Study 3 is aimed to test the role of need satisfaction in general life settings.

## Study 3

### Method

#### Procedure

Study 3 followed the same ethical rules as Study 1 and Study 2. We used a cross-lagged longitudinal design with 4 weeks between the two observations and a sample of Chilean working adults. At the baseline (September 2016; T1), participants were invited online to take part in a research project including the core measures for the present paper (job satisfaction, life satisfaction and need satisfaction *in life*^5^). Participants were informed that the project was part of a longitudinal study and were asked for their consent to participate in future waves. All T1 participants were sent a further email in October 2016 (T2). Thus, 4 weeks later (October 2016; T2), the same participants were asked to complete another online survey with identical measures. Respondents were sent an introductory email containing a brief description of the study, along with a web link to the survey created using Qualtrics software.

We followed the same rules as in Study 1 for collecting T1 and T2 data. Both our cross-sectional data at T1 (*N* = 703) and T2 (*N* = 263), and our longitudinal data (*N* = 258) fulfilled the required minimum sample size according to the power analysis.

Almost all constructs used in Study 3 showed acceptable distributions (George and Mallery, 2010). Skew values were acceptable for life satisfaction (T1: −1.30; T2: −1.60), job satisfaction (T1: −0.83; T2: −0.87), and need satisfaction in life (T1: −1.14; T2: −1.61). Kurtosis values were acceptable for life satisfaction (T1: 2.06; T2: 2.68), job satisfaction (T1: 0.24; T2: −0.01). However, Kurtosis values for need satisfaction in life were acceptable at T1 (1.86), but not at T2 (4.37).

#### Sample

In total, 703 Chilean working adults (43.2% female) aged from 21 to 69 years (Mean age = 38.45; *SD* = 9.59) finished the whole survey and completed our T1 measures. At T2, 263 participants (44.5% female) aged from 23 to 68 years (Mean age = 39.32; *SD* = 9.72) answered our core measures (37.41% response rate). Finally, 258 workers (44.6% female) aged from 23 to 68 years (Mean age = 39.10; *SD* = 9.63), answered both waves.

#### Measures

Job satisfaction was measured using the same single items used in Study 2. Life satisfaction was measured using the same single items used in Study 1. Need Satisfaction *in life* was measured using the items of the Need Satisfaction and Frustration scales, but in its original version, which aimed to explore life settings (Chen et al., 2014). The internal reliability of the need satisfaction scale was good, both at T1 (α = 0.90) and T2 (α = 0.93). We calculated a need satisfaction mean score by averaging its 12 life indicators.

### Results

#### Data analysis

All constructs were measured at T1 and T2. Descriptive statistics and intercorrelations for all the study variables are shown in Table 5 (cross-sectional analyses) and Table 6 (longitudinal analysis). We used AMOS 22 software (Amos Development Corporation, Florida, USA) to estimate an autoregressive cross-lagged model through path analysis (Finkel, 1995). We used full maximum likelihood estimation in all our analyses. Because this was a saturated model, the fit indices were perfect.

**Table 5:** **Descriptives and inter-correlations between all study 3 variables**.

	**Scale range**	***M***	***SD***	**1**	**2**	**3**	**4**	**5**
**TIME 1**								
1. Gender		1.44	0.50					
2. Age		38.45	9.59	−0.20^**^				
3. Life satisfaction	0–10	7.71	1.91	−0.06	0.09^*^			
4. Job satisfaction	0–10	6.69	2.40	−0.12^**^	0.18^**^	0.52^**^		
5. Need satisfaction in life	1–7	5.91	0.80	−0.05	0.07	0.72^**^	0.47^**^	
**TIME 2**								
1. Gender		1.44	0.50					
2. Age		39.32	9.72	−0.17^**^				
3. Life satisfaction	0–10	7.70	2.16	−0.14^*^	0.17^**^			
4. Job satisfaction	0–10	6.71	2.61	−0.18^**^	0.25^**^	0.66^**^		
5. Need satisfaction in life	1–7	5.90	0.91	−0.14^*^	0.18^**^	0.77^**^	0.66^**^	

* p < 0.05;

** *p < 0.01*.

**Table 6:** **Descriptives and inter-correlations between all study 3 variables, at Time 1 (T1) and Time 2 (T2) (longitudinal data)**.

	**Scale range**	***M***	***SD***	**1**	**2**	**3**	**4**	**5**	**6**	**7**	**8**
1. Gender		1.45	0.50								
2. Age		39.10	9.63	−0.17^**^							
3. Life satisfaction (T1)	0–10	7.76	1.98	−0.13^*^	0.13^*^						
4. Job satisfaction (T1)	0–10	6.74	2.47	−0.18^**^	0.22^**^	0.62^**^					
5. Need satisfaction in life (T1)	1–7	5.91	0.81	−0.14^*^	0.16^**^	0.74^**^	0.55^**^				
6. Life satisfaction (T2)	0–10	7.69	2.17	−0.15^*^	0.17^**^	0.85^**^	0.57^**^	0.73^**^			
7. Job satisfaction (T2)	0–10	6.68	2.62	−0.19^**^	0.24^**^	0.59^**^	0.75^**^	0.63^**^	0.66^**^		
8. Need satisfaction in life (T2)	1–7	5.89	0.91	−0.15^*^	0.17^**^	0.67^**^	0.53^**^	0.84^**^	0.77^**^	0.66^**^	

* p < 0.05;

** *p < 0.01*.

##### Cross-sectional analyses

First, we set up a cross-sectional model. At T1 (Supplementary Figure 3), we found that job satisfaction was significantly and positively related to life satisfaction, β = 0.52, *p* < 0.001. Therefore, H1 was supported at T1. To test H4, we included need satisfaction in the model. We allowed need satisfaction to predict both job satisfaction and life satisfaction (Supplementary Figure 4). Need satisfaction was positively associated with both life satisfaction, β = 0.47, *p* < 0.001, and job satisfaction, β = 0.72, *p* < 0.001. However, in support of H4, when need satisfaction was included in the model, the path between job satisfaction and life satisfaction decreased by almost half of its original value, β = 0.30, *p* < 0.001. At T2 (Supplementary Figure 3), we found that job satisfaction was significantly and positively related to life satisfaction, β = 0.66, *p* < 0.001. Thus, H1 was supported at T2. To test H4, we included need satisfaction in the model (Supplementary Figure 4). We found that need satisfaction was positively associated with both life satisfaction, β = 0.77, *p* < 0.001, and job satisfaction, β = 0.66, *p* < 0.001. In support of H4 at T2, when need satisfaction was included in the model, the path between life and job satisfaction decreased to almost half of its original value, β = 0.32, *p* < 0.001.

##### Longitudinal analysis

**Figure 5:**
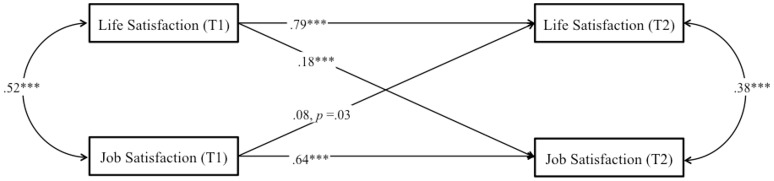
**Study 3**. Structural longitudinal model for the association between job satisfaction and life satisfaction. Coefficients shown are standardized paths. Error terms are not shown to enhance visual clarity. T1, Time 1; T2, Time 2. ^***^p < 0.001.

**Figure 6:**
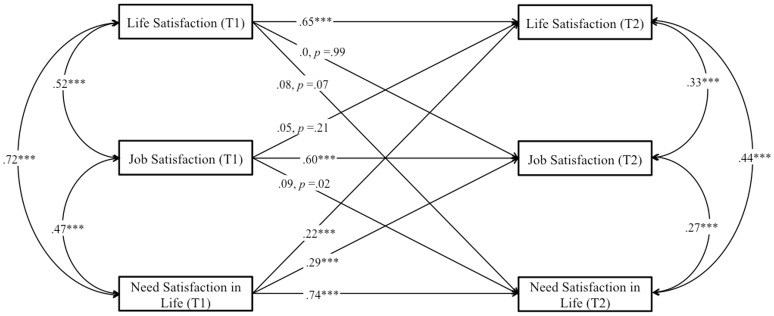
**Study 3**. Structural longitudinal model for the association between job satisfaction and life satisfaction, and need satisfaction at life. Coefficients shown are standardized paths. Error terms are not shown to enhance visual clarity. Tl, Time 1; T2, Time 2. ^***^p < 0.001.

First, we started with a structural cross-lagged reciprocal model for our core variables (job and life satisfaction), following the same strategy as for Study 2. Second, we allowed the three measures to covary within each time point, and we modeled lagged paths from each of the three measures to all three measures at each successive time point. Thus, all constructs were represented as potential antecedents and consequences of all other constructs, while controlling for stability effects. Supporting H2, we found that job satisfaction was a positive prospective predictor of life satisfaction, β = 0.08, *p* < 0.05, and that life satisfaction was a significant and positive prospective predictor of job satisfaction, β = 0.18, *p* < 0.001 (Figure 5). *R*^2^ was good for job satisfaction (0.56) and life satisfaction (0.70). Then, given the role hypothesized by need satisfaction in the job-life satisfaction relationship, we ran a structural equation model to assess whether need satisfaction influenced both variables aiming to test H4 (Figure 6). In this model, *R*^2^ was good for job satisfaction (0.60), life satisfaction (0.72), and need satisfaction (0.71). We found that need satisfaction in life was a significant and positive prospective predictor of both job satisfaction, β = 0.29, *p* < 0.001 and life satisfaction, β = 0.22, *p* < 0.001. Moreover, we found that job satisfaction was a significant and positive prospective predictor of need satisfaction in life, β = 0.09, *p* < 0.05. No other significant prospective paths were found. After controlling for need satisfaction, the reciprocal link between job satisfaction and life satisfaction became non-significant, supporting H4. We found that job satisfaction does not predict life satisfaction longitudinally, β = 0.05, *p* = 0.21 and life satisfaction does not predict job satisfaction longitudinally, β = 0.08, *p* = 0.99, when controlling for need satisfaction.

### Study 3 brief discussion

Study 3 results also replicated Study 1 findings, thus supporting H1 and H2. In addition, taking a top-down approach (Heller et al., 2002), it was found that the cross-sectional and causal bi-directional positive link between job satisfaction and life satisfaction is rooted in need satisfaction in life (Deci and Ryan, 2000). This key finding supports H4.

## General discussion

So, returning to our initial question—Are job satisfaction and life satisfaction related?—we can say that despite the limitations of previous research, three hypotheses have emerged to explain their possible association (Rain et al., 1991; Heller et al., 2002, p. 288): the segmentation, compensation, and spillover hypotheses. The segmentation hypothesis suggests that there is no association between both variables. The compensation hypothesis states that either high job satisfaction or high life satisfaction compensates for the dissatisfaction in the other area (i.e., there is a negative association). Finally, the spillover hypothesis claims that both variables are positively associated. We have tested these hypotheses through three studies involving both cross-sectional and longitudinal analysis among workers in Chile.

Consistently, Study 1, Study 2, and Study 3 showed that job satisfaction and life satisfaction are positively related both cross-sectionally and longitudinally bi-directionally, thus supporting the spillover hypothesis. Study 1 found that job satisfaction predicts increases in life satisfaction, which in turn predict increases in job satisfaction, and vice versa, thereby creating a virtuous circle in both individual and organizational well-being. Importantly, the link holds when controlling for age, gender, education, economic sector, working area, managerial functions, net monthly personal income, and net monthly family income. Thus, Study 1 aimed to approach Judge and Watanabe (1993) suggestion. He stated that when studying the link between job and life satisfaction, the use of extensive controls is crucial. Studies 2 and 3 replicated the findings of Study 1 in independent samples. Therefore, our results support the spillover hypothesis in the case of Chile and also show that this relationship is bi-directional.

However, a novel contribution of our research is the fact that the association between job satisfaction and life satisfaction may be spurious, and that a third variable could have an effect: basic need satisfaction (Deci and Ryan, 2000). Across two longitudinal studies (Studies 2 and 3), we can see that when need satisfaction is included at the same time as job satisfaction and life satisfaction, the job-life satisfaction bi-directional link becomes non-significant over time. These results were found using both a bottom-up approach (Study 2: need satisfaction *at work*) and a top-down approach (Study 3: need satisfaction *in general life settings*). This “spurious association” hypothesis has been proposed before (Heller et al., 2002; Rode, 2004), but only with regard to personality variables as mediators (e.g., Big Five, core self-evaluations). However, our research is the first attempt to test the role of the psychological needs for autonomy, competence and relatedness as stated by self-determination theory (Deci and Ryan, 2000). The results show that need satisfaction plays a key role in the process. Thus, our results support previous theorizations and findings in that a third variable may be driving the job-life satisfaction link.

Studying Chile allows us to extend previous research mostly done in the Western world. Our aim is for our results to be able to help companies to develop strategies not only for protecting their employees' mental health, but also for improving the employees' quality of life and happiness. Indeed, this issue is especially important in Chile, a country where the Ministry of Labor has recently warned that employees are in danger of serious mental health problems (Mutual de Seguridad, 2015). In addition, as mentioned previously, due the strong link between life/job satisfaction and productivity, we hope our findings may also help companies to increase their profitability and sustainability (Diener and Tay, 2012; Montero and Vásquez, 2015).

### Practical implications for people and organizations

What can be drawn from our research? Job satisfaction and life satisfaction are strongly related to several desirable outcomes both in life and at work (Diener and Tay, 2012). For example, whereas job satisfaction has been associated with higher job performance, organizational citizenship behavior and life satisfaction and lower absenteeism and turnover intentions (Heller et al., 2002; Erdogan et al., 2012), life satisfaction has been associated with higher career satisfaction, organizational commitment and job satisfaction (Diener and Tay, 2012). Our results show a key mechanism which companies may use if they wish to have happier and more engaged and productive workers: organizations should help employees to satisfy their psychological needs for autonomy, competence, and relatedness. Further, companies should help workers to feel that their behavior is volitional and meaningful (autonomy satisfaction); that they are effective and efficient in their behavior (competence satisfaction) and feel connected, appreciated and understood by important others (relatedness satisfaction) as stated by self-determination theory (Deci and Ryan, 2000; Van den Broeck et al., 2016).

Therefore, helping workers to feel that they are able to decide what to do, as well as feeling good at daily activities, and having meaningful and deep relationships with people who is important for them, are key nutrients for satisfying their psychological needs, making them more satisfied not only with their lives, but also with their jobs (Deci and Ryan, 2000; Van den Broeck et al., 2016). Thus, when companies help employees to satisfy their needs for autonomy, competence and relatedness, organizations might start a virtuous circle of flourishing both in employees' lives and at work. However, a vicious circle is also possible. If employees feel low need satisfaction, or even worse, feel that their psychological needs are frustrated (Unanue et al., 2014; Van den Broeck et al., 2016), companies may start a dangerous circle of employees' unhappiness.

Despite previous arguments supporting the importance of need satisfaction, recent research by Di Fabio and colleagues (Di Fabio and Palazzeschi, 2015; Di Fabio and Kenny, 2016a,b) has highlighted the importance of other employees' psychological resources (life resources and job resources) for promoting both job satisfaction and life satisfaction. Di Fabio and Kenny (2016a) recognize that decent work and well-being require the needs for “power, relationship, and self-determination” to be satisfied (similarly to self-determination theory postulates). However, there are a set of additional flexible life and work skills that also need to be developed. In work settings, for example, career management and self-management skills are crucial (e.g., maintaining their employability, intentionality, life-long learning, autobiographical reasoning, meaning-making, and building resilience). In life settings, for example, the development of self and reflexivity are additional key elements in this process. Further, the authors developed the *Positive Self and Relational Management* (PS&RM) model and found that Positive Lifelong Life Management (e.g., hedonic and eudaimonic well-being), Positive Lifelong Self-Management (e.g., individual level resources and self-insight in the work context), and Positive Lifelong Relational Management (e.g., relational adaptation in work and life) are the key resources that people should develop for happier lives and jobs. Other psychological resources for protecting and promoting well-being have been explored recently. For example, Di Fabio and Kenny (2016b) has shown that trait of emotional intelligence is associated with higher life satisfaction, above and beyond fluid intelligence and personality traits. In addition, Di Fabio and Palazzeschi (2015) have explored the role of resilience (among other variables): they show that resilience is associated with higher life satisfaction above and beyond fluid intelligence and personality trait.

All the skills mentioned previously draw upon psychological resources, and when developed, could help employees to foster their well-being both in life and at work. This process would complement the powerful role of need satisfaction. Thus, based on previous findings, future perspectives for intervention could focus not only on the satisfaction of the needs for autonomy, competence and relatedness, but also on developing career management and self-management skills, as well as emotional intelligence and resilience. A few programs have emerged recently in Chile for dealing with the promotion of well-being in the workplace. For instance, the Universidad Adolfo Ibáñez has launched a Graduate Program in Organizational Happiness, the *Diploma en Felicidad Organizacional* (DFO; UAI, 2016). The DFO has become a successful program aimed at training managers and consultants in the business skills and psychological resources needed for promoting well-being both in their lives and in their companies. In addition, the Chilean Ministry of Labor has developed an instrument for measuring mental health risks in the work place, called *Protocolo de Vigilancia de Riesgos Psico-sociales* (MINSAL, 2017). Depending on the companies' results with regard to employees' mental health, companies could be forced by law to implement programs to protect their employees' happiness.

### Limitations and future research

We should acknowledge some limitations in our research.

First, all our measures are self-reported, which increases the risk of common-method bias. However, self-report measures are justifiable when studying constructs that are self-referential, such as job satisfaction, life satisfaction and need satisfaction (Van den Broeck et al., 2016). In addition, we put in place several a priori controls (Podsakoff et al., 2003; Conway and Lance, 2010) in order to mitigate common-method bias: protecting respondent anonymity, explaining that there would be no right or wrong answers, and allowing participation at the location of the respondents' choice (home, office, etc.). Nonetheless, future research could consider using an additional source of data, such as asking “significant others” to triangulate information, although previous research (Heller et al., 2002) has shown that self-report measures of job satisfaction and life satisfaction do not differ significantly from measures collected from “significant others,” which gives support to the use of self-reported information in our three studies.

Second, following our first concern, the use of self-reported data may be problematic due to shared method variance. However, in our longitudinal analyses, we modeled stability paths from all T1 to all T2 measures. This procedure helps to avoid the possibility that shared method variance might inflate the cross-lagged paths across time. Third, given that the great majority of our participants were all university graduates, we should be cautious about generalizing these findings to poorer and less educated groups. Fourth, and finally, despite the strong evidence showing that job satisfaction is a temporal antecedent of life satisfaction and vice versa—and that need satisfaction explains this link, our longitudinal design still does not rule out the possibility of a third, different, unmeasured variable that influences both constructs.

Importantly, despite the previous limitations, our results considerably strengthen the case not only for a causal path from job satisfaction to life satisfaction, but also for the confounding role of basic need satisfaction in the link mentioned. We presented the first research to date in a Latin American context, showing that job satisfaction and life satisfaction are reciprocally, positively and prospectively linked to each other, but that the link may be spurious due to the important role played by need satisfaction.

## Author contributions

All authors listed have made substantial, direct and intellectual contribution to the work. WU, MG, DC, JO and AM conceptualized the study, chose the theoretical framework and measures. WU, MG and DC designed overall study. All authors wrote several sections of the initial draft, carried out analysis and interpreted results. All authors wrote, read and revised the final paper and approved it for publication.

## Funding

WU thanks the Chilean Comisión Nacional de Investigación Científica y Tecnológica. Studies 1, 2, and 3 are part of a series of papers funded by the Chilean Fondo Nacional de Desarrollo Científico y Tecnológico (Fondecyt Iniciacion) Project N^o^ 11160389.

### Conflict of interest statement

The authors declare that the research was conducted in the absence of any commercial or financial relationships that could be construed as a potential conflict of interest.

## Footnotes

^1^It is important to mention that Haar et al. (2014) explored work-life balance and several well-being outcomes across seven cultures (Malaysian, Chinese, New Zealand Maori, New European, Spanish, French, and Italian). However, despite measures of job satisfaction and life satisfaction being included, the authors did not test the associations between both constructs. In addition, Morrison et al. (2011) explored the relationship between national satisfaction and several specific well-being indicators (e.g., job satisfaction and life satisfaction) among a sample representing 95% of the world's adult population, yet although the authors reported the correlation between job satisfaction and life satisfaction (0.26), they did this to the aggregate level. Thus, they did not show the results for every single country. In addition, only the correlation between the constructs was shown, and no suitable statistical model was tested. Therefore, causality may not also be implied.

^2^Other measures (e.g., positive/negative emotions, happiness, trust, materialism, etc.) were collected as part of a broader survey on *soccer and happiness* (Unanue et al., 2017), but they are not relevant to the present research. For that study we collected data from working and non-working adults. However, for the present one, we only used data from employees in Chile. The same applies to Study 2 and Study 3.

^3^Some demographic data for participants who stayed in T1 and T2 may differ between T1 and T2 due to changes in working status between the two waves. The same applies to Study 2 and to Study 3.

^4^We also collected measures of need frustration, materialism, work outcomes (engagement, burnout, etc.), gratitude and beneficence, but they are not relevant to the present research.

^5^We also collected measures of flourishing, pro-social behavior, beneficence, work outcomes (engagement, burnout, etc.), and gratitude, but they are not relevant to the present research.
