# Supplementary material for: Revisiting the Link between Job Satisfaction and Life Satisfaction: The Role of Basic Psychological Needs
Source: Front Psychol. 2017 May 9;8:680. doi: 10.3389/fpsyg.2017.00680 (PMC5423407; doi:10.3389/fpsyg.2017.00680)
Supplement: Supplementary file 1 [file DataSheet1.docx]

***Supplementary Material***

**Revisiting the Link Between Job Satisfaction and Life Satisfaction: The Role of Basic Psychological Needs**

**Wenceslao Unanue*, Marcos Gómez, Diego Cortez, Juan Carlos Oyanedel, Andrés Mendiburo.**

*** Correspondence:** Corresponding Author: wenceslao.unanue@uai.cl


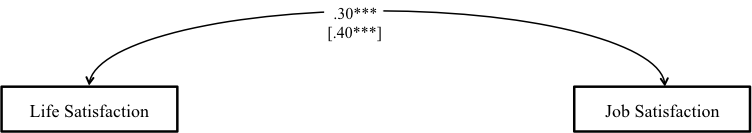


**Supplementary Figure 1.** Study 2. Structural correlational model for the association between Job Satisfaction and Life Satisfaction at T1 and T2. T2 data are in brackets. Coefficients shown are standardized paths. T1: Time 1, T2: Time 2. *** p < .001.


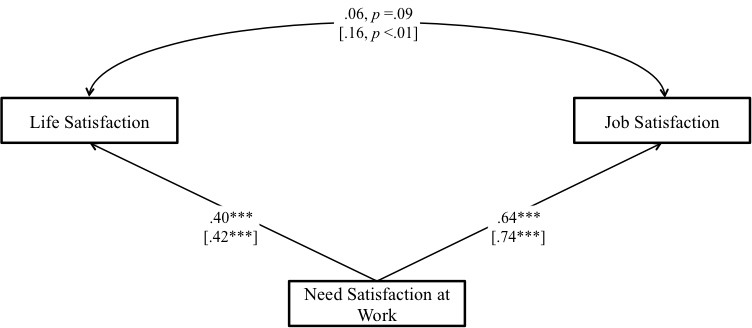


**Supplementary Figure 2.** Study 2. Structural correlational model for the association between Job Satisfaction and Life Satisfaction at T1 and T2. T2 data are in brackets. Coefficients shown are standardized paths. T1: Time 1, T2: Time 2. *** p < .001


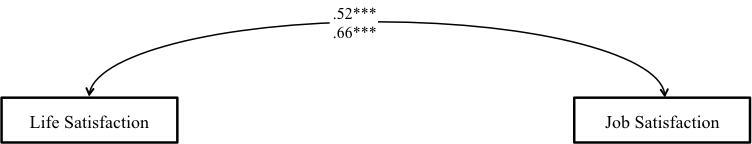


**Supplementary Figure 3.** Study 2. Structural correlational model for the association between Job Satisfaction and Life Satisfaction at T1 and T2. T2 data are in brackets. Coefficients shown are standardized paths. T1: Time 1, T2: Time 2. *** p < .001


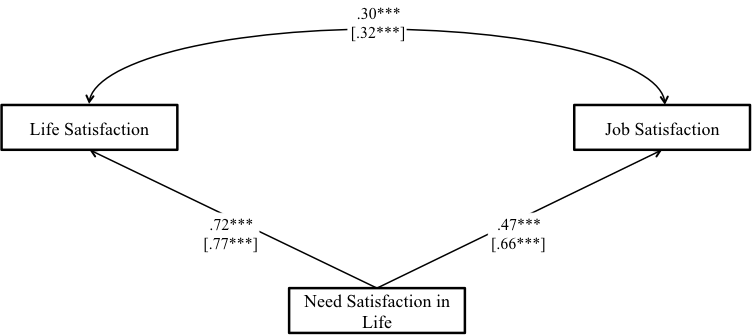


**Supplementary Figure 4.** Study 2. Structural correlational model for the association between Job Satisfaction, Life Satisfaction and Need Satisfaction at Life, at T1 and T2. T2 data are in brackets. Coefficients shown are standardized paths. T1: Time 1 and T2: Time 2. *** p < .001
